# Report of an Unusual Case of Pneumonia

**Published:** 1910-05

**Authors:** J. E. Sommerfield

**Affiliations:** Atlanta, Ga.


### REPORT OF AN UNUSUAL CASE OF PNEUMONIA.

J. E. SOMMERFIELD, M. D., ATLANTA, GA.

Those of us who are called upon to treat a patient having Broncho-Pneumonia, realize how seriously ill such a patient is and how dubious is the prognosis, when we find the patient with an anxious expression of the countenance, high fever, cough, dyspnœa, pulse 100-120, respiration 40-50 per minute and the patient struggling for life. Fortunately for us, in the majority of such cases, resolution soon sets in, the pulse and respirations return to the normal and our patient is on the high road to recovery.

If a patient with a history as given above is a cause of anxiety, how much more worried must we be when we find a patient with all these symptoms greatly exaggerated and this grave condition continuing for some time. As the case to be reported ended in recovery, we felt rewarded for the worry and time spent. I trust that the report will interest the members of this Association.

Sarah B., age 3½ years, with a negative family history, had never been sick until the early spring of 1909, when she and all her brothers and sisters had whooping-cough. The attack was comparatively mild and lasted about five weeks, leaving her with a hacking cough which persisted about three months. On October 6th, the mother noticed that the child coughed, but as it seemed to be slight and did not inconvenience the child nor keep her from her play, no attention was given to same. She was put to bed at her usual hour, apparently well. During the night the parents were awakened by her cough and her rapid and difficult breathing. They attempted to arouse her, but met with poor success. I saw her shortly afterward and found her in the following condition:

She was in a stupor from which she could only be aroused with difficulty, cyanotic, dyspnœa quite marked—pulse 160, respirations 100 per minute—temperature (rectal) 103°. Examination showed throat normal. Chest on percussion, small areas of dullness both anteriorly and posteriorly—on auscultation medium and fine moist vales over entire chest. Abdomen highly tympanitic. Diagnosis: Broncho-Pneumonia.

A hypodermic injection of 1-100 grain of nitrate of strychnine was given at once. This relieved the cyanosis somewhat, but had little or no effect upon pulse and respirations.

Consultation was asked for and granted. The consultant suggested the possibility of membranous croup, so a culture test was made from the throat, but with a negative result. At my suggestion, the child was removed to a private hospital as the mother was unable to give her the proper attention and besides, three other children slept in the same room. For nearly two weeks the exhausting rapid pulse and respiration continued, the respirations rarely going below 90 per minute. The tympanitic condition was also the cause of much worry and frequent turpentine and asafœtida enemas gave some relief. After two weeks the chest began to clear up—the respirations and pulse became less frequent and at the end of four weeks the patient was discharged with a pulse of 90 and 22 respirations per minute. The treatment was as follows:

1-100 grain of strychnine nitrate and 1-500 grain of Atropine were given every four hours—inhalations of oxygen given at frequent intervals. A cough mixture containing Terpin hydrate, Heroin and Cascara was taken for some time.

About six weeks after her discharge I was called one midnight to see her, as she had fever, coughing, complained of pain in her chest and her respirations were sixty per minute. An expectorant quickly relieved her and in the course of four or five days she was discharged. I saw her several days ago and her mother stated that she had remained entirely well since her last illness.

